# Evaluating the efficacy of photoplethysmography-derived stiffness index, sine-waveform ratio, and heart rate variability total power for cardiovascular health assessment

**DOI:** 10.1186/s12872-026-05914-6

**Published:** 2026-05-09

**Authors:** Lei Wang, Meng-Yu Hsiao, Zi-Jun Chen, Ruo-Jhen Wu, Meng-Ting Wu

**Affiliations:** 1 Department of Electrical Engineering, Feng Chia University, Taichung, Taiwan; 2 Division of Neurosurgery, Department of Surgery, Cheng-Hsin General Hospital, No. 45, Zhenxing St, Beitou Dist, Taipei City, 112401 Taiwan (R.O.C.); 3 Department of Neurological Surgery, Tri-Service General Hospital, National Defense Medical Center, Taipei, Taiwan

**Keywords:** Photoplethysmography, Cardiovascular disease, Arterial stiffness, Sine-waveform, Heart rate variability, Preliminary assessment

## Abstract

**Introduction:**

This study aimed to predict risk of cardiovascular disease (CVD) in participants unaware of their cardiovascular health using a convenient approach.

**Methods:**

Continuous 100-second photoplethysmography (PPG) signals were recorded from 144 participants. The subjects were subsequently categorized based on the presence or absence of CVD. The proportions of abnormal stiffness index (SI), the sine-waveform ratio (SIN ratio), a morphological index of PPG waveform regularity used as a marker for cardiovascular pathological aging, and total power (TP), derived from heart rate variability (HRV) frequency-domain analysis to reflect autonomic nervous system activity, were compared.

**Results:**

A total of 144 participants were enrolled in this study (CVD group, *n* = 54; healthy control group, *n* = 90). CVD group had higher SIN ratio (37.4 ± 27.8% vs. 21 ± 23.9%) and lower TP (1587.0 ± 262.9 vs. 1804.7 ± 261.7ms²) compared to healthy controls (*p* < 0.001). CVD group had higher rates of abnormal SI (46% vs. 24%) and SIN ratio > 40% (42.6% vs. 23.5%), all *p* < 0.01. The combination of abnormal SI values, SIN ratio > 40%, and TP < 1500 ms² indicated a high likelihood of CVD (*p* < 0.001).

**Conclusion:**

PPG-derived SI, SIN ratio, and TP are valuable indicators that are potentially associated with cardiovascular health. Our results provided a convenient, inexpensive, and automated approach for preliminary assessment of the presence of CVD. Further large-scale studies are needed to validate these findings.

**Supplementary Information:**

The online version contains supplementary material available at 10.1186/s12872-026-05914-6.

## Introduction

Cardiovascular disease (CVD) is the leading cause of death worldwide [1]. CVD prevalence increases with each decade of life, with higher prevalence among men than women [2]. CVD is a major source of direct and indirect health care costs [2]. Many patients do not have any CVD symptoms before the first serious cardiovascular (CV) event (e.g., myocardial infarction or stroke). Literature has reported that approximately 30% of patients presenting with acute coronary syndromes do not have a prior diagnosis of CVD; therefore, identifying asymptomatic individuals for preventive medication may reduce the risk of future CVD morbidity and mortality [3, 4]. To effectively screen and manage those at moderate-to-high-risk for CV events, measures of arterial stiffness index as markers for CVD should be incorporated into routine clinical practice. Proper stratification of these individuals can help target preventive interventions to those who are most likely to benefit, or reduce the use of preventive interventions on those who are at low risk.

Atherosclerosis is a chronic inflammatory disease of the arteries and is the key pathophysiological process underlying CVD [5]. CV events have traditionally been predicted using an algorithm that combines conventional atherosclerotic risk factors [6–8]. However, vascular biomarkers such as carotid ultrasonography, the ankle-brachial index, arterial stiffness, central hemodynamics and wave reflections, endothelial function, and circulating biomarkers related to vascular wall biology (e.g., high sensitivity C-reactive protein) are also recommended for the primary and secondary assessments of CV events [9].

**Fig. 1:**
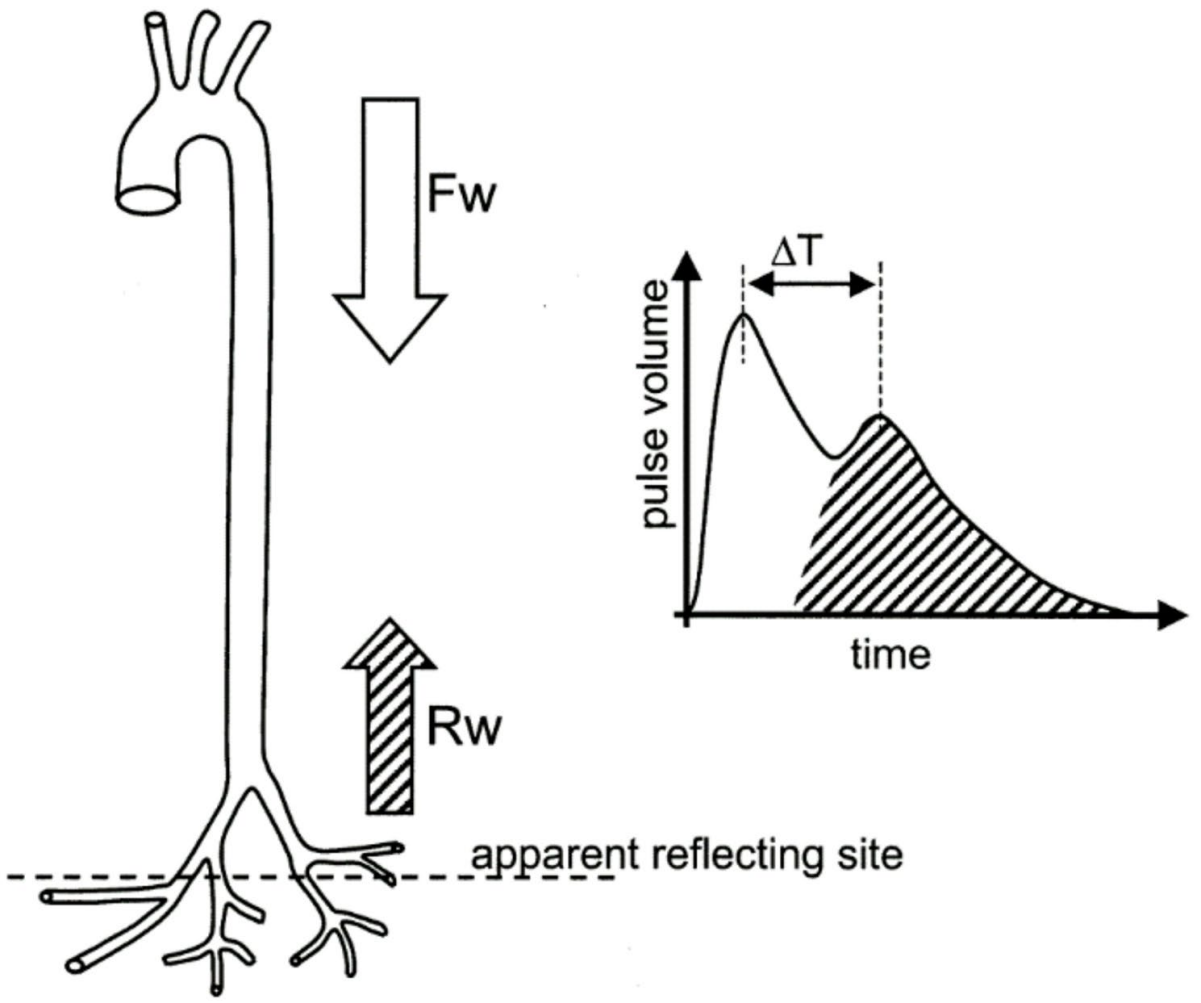
SI schematic

Photoplethysmography (PPG) is a non-invasive optical measurement technique for measuring blood volume changes with each pulse and is a promising monitoring device for simplicity and convenience of use [10, 11]. PPG signals can monitor different aspects of CV conditions, such as heart rate, blood velocity, hypertension, heart rate variability (HRV), augmentation index, and large artery stiffness index (SI) [12]. SI assesses arterial stiffness by calculating the time relationship between percussion and diastolic wave, as exemplified in Fig. 1 [13]. In our previous study, we described a modified PPG signal processing and analysis procedure to obtain an SI reflecting arteriosclerosis severity and emphasized the importance of correctly identifying sine-waveform (SIN) to prevent over- or under-estimated SI values [14].

The European consortium reported normal references and stated that 10 m/s was the cutoff for pathological values [15]. However, it is known that age, race/ethnicity, sex, family history, and lifestyle may contribute to differences in CV health [16]. In addition, physical fitness at the time of PPG measurements also affects the quality of the signals. Therefore, the present study aimed to predict CVD in patients beyond traditional risk factors using a convenient, inexpensive approach. The ability of SI and SIN marker, obtained from PPG, and total power (TP), obtained from HRV, in predicting CVD was evaluated.

## Materials and methods

### Study design and participants

This prospective study collected 100-second PPG signals from 144 participants. Volunteers were sought to participate in the testing in the most convenient environment, and the samples were primarily collected based on the following categories. Cheng Hsin General Hospital provides sampling focused primarily on the elderly, patients with a history of CVD, and medical staff who often experience fatigue due to long working hours. The CVD group in this study comprised patients with a documented history of hypertension, hyperlipidemia, and diabetes mellitus, as well as individuals who had undergone cardiac surgery. To ensure clinical relevance, this group also included patients diagnosed with stable coronary artery disease, chronic heart failure, and peripheral artery disease. Notably, patients with atrial fibrillation were excluded to focus the analysis on morphological waveform changes driven by vascular pathological aging rather than acute rhythmic irregularities. The aim was to test whether the indicators proposed in this study showed significant differences in these specific groups. Additional data were collected from administrative staff and young student volunteers from various departments of Feng Chia University to supplement the sample with healthy individuals of all ages and young people at their physical peak, serving as control groups for the results analysis. Participants with cardiac arrhythmia or implanted pacemakers were excluded from this study. This study was approved by the institutional review board (approval no.(770)109 −10), and written informed consent was obtained from all participants. All study procedures adhered to the 1975 Declaration of Helsinki.

### PPG signal preprocessing and SI value calculation

PPG signal preprocessing and SI calculation followed the procedures described in our previous study [14]. In brief, the mathematical formula for SI is given in Eq. (1), where ∆T is the time difference between the main pulse wave and the re-beat pulse, and h is the height of the subjects.

1 $${SI}={h}/\triangle{T}$$

A larger SI value indicated faster propagation speed of the diastolic phase, suggesting poor vascular elasticity. Conversely, a lower SI value indicates slower diastolic phase propagation, implying better vascular elasticity. For estimating the normal range of SI value for different ages, data from the subjects with known CVD was excluded from SI normal reference range estimation to reduce confounding. Following a previously published method [17], SI results from the remaining subjects, who had no known CVD, was regressed against age with 95% confidence interval to define the age-specific normal reference range for SI. Table 1 lists the reference values in 5-year age interval.

**Table 1:** The normal range of SI values

Age (years)	16–20	21–25	26–30	31–35	36–40	41–45	46–50	51–55
Lower limit	3.489	3.664	3.838	4.013	4.187	4.362	4.536	4.710
Upper limit	12.131	12.306	12.480	12.654	12.829	13.003	13.178	13.352

Age (years)	56–60	61–65	66–70	71–75	76–80	81–85	86–90	91–95
Lower limit	4.885	5.059	5.234	5.408	5.583	5.757	5.931	6.106
Upper limit	13.527	13.701	13.875	14.050	14.224	14.399	14.573	14.748

### Definition of SIN (with an unapparent dicrotic notch)

The dicrotic wave in a PPG signal represents a secondary upward deflection following the systolic peak. It occurs during the diastolic phase of the cardiac cycle and is associated with the closure of the aortic valve and the subsequent rebound of the blood column in the arteries. Having SIN (without dicrotic notch) appearing in the PPG signals, as shown in Fig. 1, affects the accuracy of arterial stiffness calculation. This waveform lacks a clear dicrotic peak, and using the original first derivative method to capture the sine-wave tends to locate the region close to the trough, resulting in an underestimation of SI value. To solve the problem of incorrect sine-wave capture, which could impact SI value calculation, the following methods were used to identify the presence of sine-waves in this study:

1. As there were more than two depressions on the PPG (Supplement Fig. 1a), we identified the points closest to B(t_1_), the start of the percussion wave, and B(t_2_), the wave trough of the diastolic wave, present within the timeframe. Then we adopted the third order Bézier curve equation in Eqs. (2) and (3) in order to identify two control points, P_1_ and P_2_ [18]. Among these identified points, P_0_ and P_3_ were the first lowest point of first derivative and the trough of PPG signals, respectively (Supplement Fig. 1c). Also, for reducing the range of search, an auxiliary line was drawn from P_0_ to sine-wave as indicated by the dotted line in Supplement Fig. 1a.

    

2 $$\:\mathrm{t}=\frac{\mathrm{B}\left(\mathrm{t}\right)-{P}_{0}}{{P}_{3}-{P}_{0}}\:，0\le\:\mathrm{t}\le\:1$$

3 $$\:\mathrm{B}\left(\mathrm{t}\right)={P}_{0}{(1-t)}^{3}+3{P}_{1}{(1-t)}^{2}t+3{P}_{2}\left(1-t\right){t}^{2}+{P}_{3}{t}^{3}$$

2. As indicated by Supplement Fig. 1f, when there is only one depression on the curve of distance from all points to auxiliary B(t_1_), the presence of an unapparent dicrotic notch or unapparent diastolic wave was implied. We may then adopt Eq. (4) to calculate integral B(t_2_). And subsequently, Eq. (3) was adopted to identify control points P_1_ and P_2_, in addition to the auxiliary line drawn from P_0_ to B(t_2_), as indicated by the dotted line in Supplement Fig. 1b.

 Eq. (4)

4 $$\:{\mathrm{B}(\mathrm{t}}_{2})=⌊\frac{{P}_{3}-B\left({t}_{1}\right)}{2}⌋$$

Further based on Eq. (5), we acquired the correlation between the unapparent dicrotic notch curve drawn by Bézier method and PPG waveforms. A lower correlation indicated a higher possibility of a diastolic wave being present in the PPG signal. According to our preliminary observation from 50 PPG waves, the study found it effective to identify the SIN waves in 99.5% of samples.

 Eq. (5)

5 $$\:\mathrm{r}=\frac{n\sum\:xy-\sum\:x\sum\:y}{\sqrt{n\sum\:{x}^{2}-{\left(\sum\:x\right)}^{2}}\sqrt{n\sum\:{y}^{2}-{\left(\sum\:y\right)}^{2}}}$$

In this study, the proportion of SIN in PPG signals was defined as the SIN ratio. Specifically, the SIN ratio functions as a morphological indicator of PPG regularity, offering a unique perspective on CV pathological aging that complements traditional stiffness metrics. A higher SIN ratio indicated a higher proportion of SIN. Since over 70% study subjects had SIN ratio less than 20% and most of the higher values were observed in subjects over 45 years of age, an abnormal SIN ratio was defined as > 40% according to the distribution of measurements.

### HRV data collection and frequency-domain HRV indices calculation

HRV data for 100 subjects was first obtained from the MIMIC database and the HRV indices for 100 s of data were compared to those obtained from a standard 5-minute measurement. We observed that the trends in TP values from 100-second data were similar to those from the 5-minute data, validating the use of 100-second data rather than a longer 5-minute measurement (Supplement Fig. 2) for quick and efficient HRV measurement, suitable for rapid assessment in practical settings. For each participant, the HRV data was collected from continuous PPG signals recorded for 100 s. The collected HRV data were normalized. After normalization, the heart interval data was subjected to a discrete Fourier transform to obtain the power spectral density graph. For this study, TP was calculated from the spectrum ranging from 0 Hz to 0.4 Hz to assess the overall HRV of the subject.

Since 75% of the test subjects with TP below 1500 ms^2^ were CVD patients or those who self-indicated to be in a state of fatigue at the time of the test, and almost 44% of CVD patients had TP value below 1500, a TP value below 1500 ms² was set as the threshold for abnormal heart rhythm variability in this study.

### Logistic regression analysis

Multivariate analyses were conducted to delineate the role of the proposed PPG-derived markers in predicting CVD, adjusting for potential confounding from age, sex, and anthropometrics.

## Results

### Patient demographics

A total of 144 participants were enrolled in this study, comprising 58 males (40.3%) and 86 females (59.7%). The subjects’ mean age was 48.6 ± 20.4 years. Within the CVD group (*n* = 54), the average age was 63.6 ± 13.0 years, with a sex distribution of 46.3% male and 53.7% female. In contrast, the healthy control group (*n* = 90) had an average age of 39.6 ± 18.6 years. As such, patients with CVD (*n* = 54) were older (63.6 ± 13.3 vs. 39.6 ± 18.6 years old) and heavier in weight (68.1 ± 15.4 vs. 60.1 ± 11.6 kg) (all *p* < 0.001) than those without CVD (*n* = 90) (Table 2). Compared to the group without CVD, all HRV parameters were lower in the CVD group, (TP: 1587.0 ± 262.9 vs. 1804.7 ± 261.7, low frequency: 208.6 ± 112.5 vs. 279.4 ± 111.8 and high frequency: 101.5 ± 49.2 vs. 169.4 ± 75.1), all *p* < 0.001. However, it is worth noting that the SI value was similar between CVD (9.0 ± 3.0) and no CVD (8.7 ± 2.5), *p* = 0.585 (Table 2). This suggested that other factors besides SI value should be used to help screen for the presence of CVD.

**Table 2:** Demographics and clinical characteristics by presence of CVD

	All (*N* = 144)	CVD (*n* = 54)	No CVD (*n* = 90)	*P* value
Female	86 (59.7%)	29 (53.7%)	57 (63.3%)	0.254^#^
Age (years), mean ± sd	48.6 ± 20.4	63.6 ± 13.0	39.6 ± 18.6	< 0.001^‡^
Height (cm), mean ± sd	162.6 ± 8.8	160.9 ± 9.4	163.6 ± 8.2	0.069^†^
Weight (Kg), mean ± sd	63.2 ± 13.7	60.1 ± 11.6	68.1 ± 15.4	< 0.001^‡^
Comorbidity, n (%)				
Hypertension	38 (26.4%)	38 (70.4%)	0	
Hyperlipidemia	21 (14.6%)	21 (38.9%)	0	
Hyperglycemia	11 (7.6%)	11 (20.4%)	0	
Drinking				0.626^
No	105 (73.4%)	39 (72.2%)	66 (74.2%)	
Quitted	5 (3.5%)	3 (1.9%)	2 (2.2%)	
Sometimes	5 (3.5%)	1 (1.9%)	4 (4.5%)	
Yes	28 (19.6%)	11 (20.4%)	17 (19.1%)	
Regular Exercise				0.212^#^
No	71 (49.3%)	23 (42.6%)	48 (53.3%)	
Yes	73 (50.7%)	31 (57.4%)	42 (46.7%)	
Anxiety				0.122^
No	118 (83.1%)	40 (75.5%)	78 (87.6%)	
Maybe	4 (2.8%)	2 (3.8%0	2 (2.2%)	
Yes	20 (14.1%)	11 (20.8%)	9 (10.1%)	
PPG measurements				
SI value	8.8 ± 2.7	9.0 ± 3.0	8.7 ± 2.5	0.585^‡^
SIN (%)	27.1 ± 26.5	37.4 ± 27.8	21.0 ± 23.9	<0.001^‡^
HRV				
TP	1723.1 ± 281.8	1587.0 ± 262.9	1804.7 ± 261.7	< 0.001†
HF	252.8 ± 116.8	208.6 ± 112.5	279.4 ± 111.8	< 0.001^‡^
LF	143.9 ± 74.1	101.5 ± 49.2	169.4 ± 75.1	< 0.001^‡^

LF, HF, and TP are measures of HRV. SIN represent the presence of an unapparent dicrotic notch on PPG

*Abbreviations*: *CVD *cardiovascular disease, *HF *high frequency, *HRV *heart rate variability, *LF *low frequency, *SI *stiffness index, *SIN *sine-waveform, *TP *total power

#Chi square test, ^fisher’s exact, †Student’s t, ‡Mann-Whitney U

### Improvement of SIN identification and SIN ratio

Table 3 shows the proportion of participants with abnormal SIN ratios before and after applying the modified methods to identify the presence of SIN. The proportion of participants with abnormal SIN (SIN > 40%) was indicated for each age group. The modified method identified more participants with SIN > 40% for age groups 41–50 (from 0 to 11.1%), 51–60 (10.0% to 33.3%), 61–70 (30.4% to 50.0%), and 71–80 (20% to 60%). The younger (1–40yrs) (4.8% to 4%) and elder > 80 years (80%) age groups were less affected by the method of SIN detection.

**Table 3:** The proportion of participants with abnormal SIN ratios (SIN > 40%) before and after applying the modified methods to identify the presence of SIN by age group

Age Group (years)	Abnormal SIN, Before (*n* = 80)	Abnormal SIN, After (*n* = 144)
1–40	1 (4.8%)	2 (4.0%)
41–50	0 (0.0%)	2 (11.1%)
51–60	1 (10.0%)	10 (33.3%)
61–70	7 (30.4%)	13 (50.0%)
71–80	3 (20.0%)	9 (60.0%)
> 80	4 (80.0%)	4 (80.0%)

SIN represent the presence of an unapparent dicrotic notch on PPG

*Abbreviation: SIN* sine-waveform

**Fig. 2:**
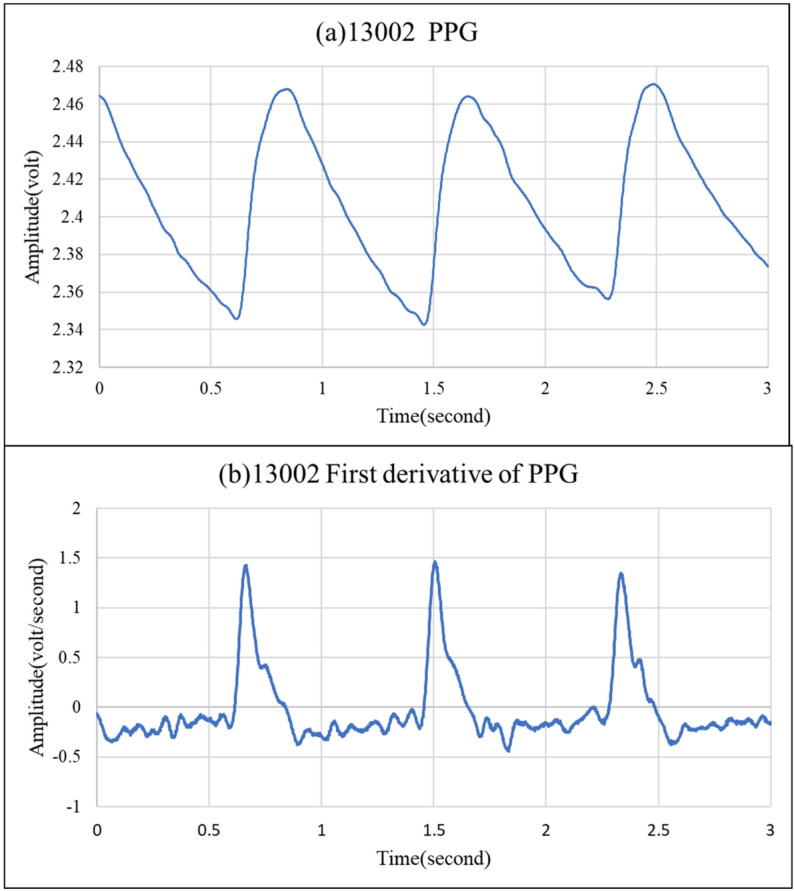
A representative PPG signal segment from participant No.13002 (**a**) and the first derivative PPG (**b**)

A representative illustration from PPG signals of a participant (Case 13002, aged 74) with inaccurate SI calculation is shown in (Fig. 2). The waveform clearly demonstrated a SIN wave without dicrotic notch. The SI value for this participant was 8.11 m/s (below the normal range for this age group); however, the SIN ratio was calculated as 18%. It is apparent from the figure that there is a detection error and the SI value is likely inaccurate. After applying the improved SIN identification method, the new SI value was calculated as 10.5 m/s, and the SIN ratio was 94%, which corresponds to the image captured, representing the true arterial status.

### Indicators for CVD

#### SI abnormality

Table 4 presents the number of participants with abnormal SI values by age group according to the established SI reference for each age group. Participants were further grouped into those with and without CVD. It was observed that the proportion of participants with abnormal SI values above 50 years of age (38–65%) was higher compared to those aged < 50 years (16–17%). Moreover, individuals with CVD show higher proportions of abnormal SI values (23–60%) compared to those without CVD (5–20%). Overall, 46% of participants with CVD have abnormal SI values, compared to those without CVD (24%). Further comparison of average SI values between participants with and without CVD shows that the average SI value was 10.03 m/s for those with CVD and 8.84 m/s for those without, indicating that individuals with CVD have higher SI values than normal individuals.

**Table 4:** The percentage of participants with abnormal SI values, stratified by cardiovascular diseases and age groups

	Abnormal SI		
	CVD, *n* (%)	No CVD, *n* (%)	Total, *n* (%)
Age Group (years)			
1–40	-	12 (16%)	12 (16%)
41–50	-	3 (17%)	3 (17%)
51–60	7 (23%)	5 (20%)	12 (43%)
61–70	6 (23%)	4 (15%)	10 (38%)
> 71	12 (60%)	1 (5%)	13 (65%)

*Abbreviations*: *CVD *cardiovascular disease, *SI *stiffness index

#### SIN ratio

As shown in Table 5, the proportion of participants exceeding the normal reference value for SIN was higher among participants with CVD compared to those without. 42.6% of participants with CVD had abnormal SIN, when abnormal SIN ratio was defined by > 40%. Among all participants with SIN vale > 40%, 57.5% had CVD. When the definition of abnormal SIN ratio was defined as > 80%, there was one patient without CVD (14.3%). This participant was found to be underweight and had comorbid conditions of renal and hepatic cysts upon case review. This finding suggested that individual medical conditions may also affect PPG measurement results. Overall, in this study, individuals with a SIN ratio > 80% had an 85.7% probability of having CVD, highlighting the importance of the accurate identification of SIN waves and ratios for assessing arterial stiffness.

**Table 5:** Percentages of participants with abnormal SI values, SIN ratios, and TP stratified by with or without cardiovascular diseases

	CVD (*n* = 54)	No CVD (*n* = 90)
SIN > 40%	23 (42.6%)	17 (18.9%)
SIN > 80%	6 (11.1%)	1 (1.1%)
SI abnormal + SIN > 40%	15 (27.8%)	8 (8.8%)
SI abnormal + SIN > 80%	3 (5.6%)	0 (0%)
SI abnormal + SIN > 40% + TP < 1500 ms²	8 (14.8%)	0 (0%)
SI abnormal + SIN > 80% + TP < 1500 ms²	1 (1.9%)	0 (0%)

SIN represent the presence of an unapparent dicrotic notch on PPG. TP is a measure of heart rate variability

*Abbreviations*: *CVD *cardiovascular disease, *SI *stiffness index, *SIN *sine-waveform, *TP *total power

The proportion of participants with abnormal SI values + abnormal SIN (SIN ratio > 40%) in the CVD group (27.8%) was higher those without CVD (8.8%) **(**Table 5). 65% of participants with abnormal SI values + SIN ratios > 40% were likely to have CVD, and when abnormal SIN was defined as SIN ratio > 80%, all participants (100%) with abnormal SI values + abnormal SIN have CVD. This suggested that SI value and SIN ratio are useful in evaluating CVD **(**Table 5**)**.

### Influence of physical fitness

**Fig. 3:**
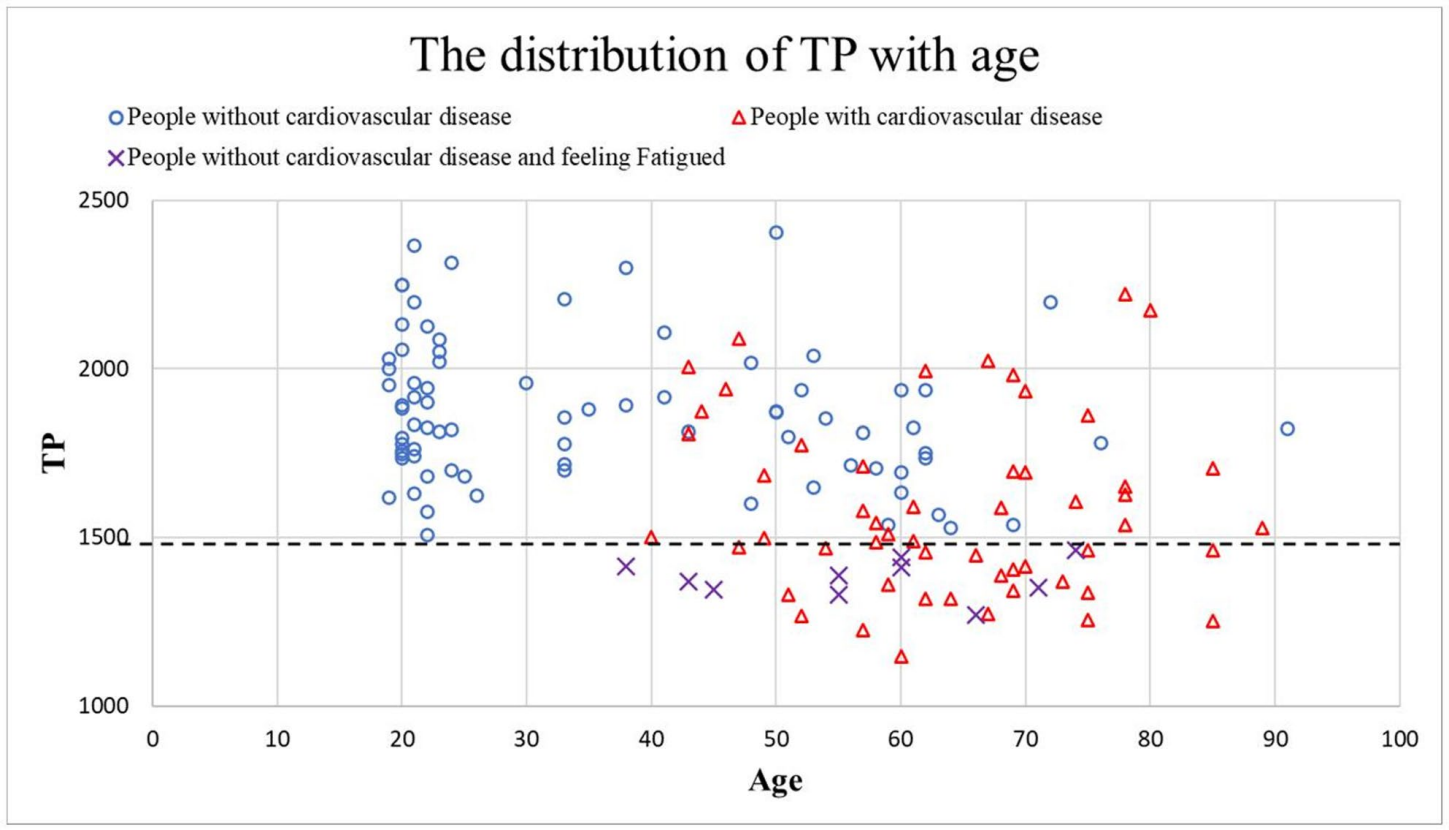
Distribution of TP Values by age. Individuals with cardiovascular disease were denoted △, without cardiovascular diseases were denoted O, without cardiovascular disease and reported fatigued were denoted X. The reference line for abnormal TP was TP values < 1500 ms²

TP values and age did not increase proportionally (Supplement Table 1). However, the percentage of participants with CVD and TP values below normal (TP < 1500 ms^2^) was higher than that of individuals without CVD. Figure 3 shows the distribution of TP values by age. It was observed that participants with TP values < 1500 ms² were mostly in the age range of 50–80 years, although the proportion of TP < 1500 ms² did not increase proportionally with age. The majority (71%) of participants with TP values < 1500 ms² were in the CVD group (denoted △ in Fig. 3). 29% of participants with TP values < 1500 ms², were without CVD (29%) (denoted X in Fig. 3), and these participants reported fatigue when the measurements were taken. The reasons for fatigue reported included overtime work and poor sleep quality. Excluding participants with reported fatigue, all other individuals with TP values < 1500 ms² have confirmed CVD.

Combining the three indicators investigated, individuals with abnormal SI values, SIN ratio > 40%, and TP < 1500 ms², were almost certain to have CVD. Using these criteria no individuals without CVD were falsely identified as having CVD. Individuals with abnormalities in any two of the three indicators have a 63% chance of having CVD, and 76% of CVD patients exhibit abnormal values in one to three indicators, indicating signs of arterial stiffness.

### Multivariate logistic regression analysis

Statistical analysis revealed highly significant differences (*p* < 0.001) between the CVD and healthy control groups across parameters including age, weight, SIN ratio, and various HRV metrics (TP, HF, LF) (Table 2). However, while these results highlight distinct physiological profiles, univariate testing fails to account for confounding factors such as age or multicollinearity between variables. Notably, the SI value alone showed no significant difference between groups (*p* = 0.585), suggesting that arterial stiffness as a standalone metric possesses limited preliminary assessment utility. Consequently, our multivariate logistic regression underscored the superior predictive power of calibrated TP < 1500 ms^2^ and the “SI + SIN + TP” composite indicators. These findings suggest that while SIN ratio correlates with age, its true clinical value lies in its role as a ‘precision filter’ for SI, significantly enhancing diagnostic specificity and overcoming the inherent limitations of single-parameter assessment.

The results shown in Table 6 demonstrate that abnormal SI (value > age-specific upper limit) (adjusted OR = 4.42, *p* = 0.04) and TP < 1500 ms^2^ (adjusted OR = 13.22, *p* = 0.005) are significantly and independently associated with the occurrence of CVD. These results suggest that SI and TP serve as more potent predictors of disease status that are independent of age. This statistical evidence confirms that the observed changes in PPG waveforms do not merely reflect the natural aging process, but rather represent the intrinsic CV pathological state of the participants. Furthermore, positioning SIN ratio > 40% as an enhancing factor significantly improves diagnostic specificity. Furthermore, the extreme threshold of SIN > 80% proved to be a highly specific marker (adjusted OR = 11.10, *p* = 0.008). The triple-indicator models (composite A: abnormal SI + SIN > 40% + TP < 1500 ms^2^; composite B: abnormal SI + SIN > 80% + TP < 1500 ms^2^) demonstrated a diagnostic power superior than single factors, with an adjusted OR of 33.82 (*p* < 0.001) and 61.87 (*p* = 0.035), respectively. These results suggest that multi-parametric PPG analysis, particularly using calibrated thresholds and composite indicators, provides a far more accurate assessment of CV pathological states than single-metric evaluations.

**Table 6:** Multivariate logistic regression analysis for CVD predictors

Predictors	*p*-value	aOR	95% CI
SI	0.422	1.05	(0.92, 1.20)
Abn SI	0.040*	4.42	(1.07, 18.25)
TP	0.317	0.99	(0.99, 1.00)
Abn TP < 1500 ms^2^	0.005*	13.22	(2.22, 78.96)
SIN > 40%	0.572	0.68	(0.18, 2.59)
SIN > 80%	0.008*	11.10	(1.89, 65.21)
Composite A (Abn SI + SIN > 40% + TP < 1500 ms^2^)	< 0.001*	33.82	(4.12, 277.5)
Composite B (Abn SI + SIN > 80% + TP < 1500 ms^2^)	0.035*	61.87	(1.28, 298.4)

SIN represent the presence of an unapparent dicrotic notch on PPG. TP is a measure of HRV

*Abbreviations*: *Abn *abnormal, *aOR* adjusted odds ratio, *CI *confidence interval, *CVD *cardiovascular disease, *SI *stiffness index, *TP *total power, *SIN *sine-waveform

* *p* < 0.05 is considered statistically significant. The multivariate logistic model adjusted for age, sex, and body mass index

## Discussion

This study utilized PPG for a fast and convenient approach to assess the risks of CVD. Moreover, an improved method for identifying the presence of sine-waveform allowing more accurate preliminary assessment for arterial stiffness and CVD was proposed. The three indicators were SI value, SIN ratio > 40 or 80%, and TP < 1500 ms².

Arterial SI is an independent CV risk factor and a predictor of all-cause mortality [19], which increases with age [20], resulting in increased pulse wave velocity (PWV)-linked arterial stiffness [21]. Consequently, PPG-based approaches for assessing arterial stiffness index are often designed to include the aortic pathway in PWV measurements [22]. As described in our previous study [14], three PPG signal parameters, including S-value, systolic peak-to-diastolic peak time difference, and the percentages of SINs, were used to assess and preprocess the signal quality used in the present study. This was necessary to identify individuals with mixed PPG waveforms and unapparent dicrotic notch in the PPG pulse waveform to improve the subsequent accuracy of SI calculation [14]. In real practice, the identification of SIN is also complicated by non-typical SIN. Therefore, the present study further investigated methods to improve the accurate identification of SIN for a more sensitive assessment of CV health based on PPG.

Aging is associated with major physiological and psychological changes in humans. In our study, age group differences in SI value, and SIN ratio were observed. Several studies have investigated the effect of aging on PPG signal characteristics [23–25], and have examined the associations between aging and the first and second derivatives of the PPG as an arterial stiffness indication [26–28]. A study by Takazawa et al. [29] used the second derivative of the PPG as an index of vascular aging. In our present study, the auxiliary line method was used to preliminarily determine mixed PPG waveforms, atypical SINs were subjected to further analysis with additional criteria - the distance between peak and trough of first derivatives and its fluctuation in amplitude. After applying the modified methods to more accurately identify SINs, we showed that the proportion of abnormal SIN ratio (> 40%) increased with age. The improved determination method mainly affected participants aged between 40 and 80 years. A possible justification could be that the vascular health of children, and adults under 40 years old is relatively healthy, with a lower probability of having unapparent dicrotic notch compared to older other age groups. On the other hand, participants over 80 years old tended to have issues with vascular stiffness, likely resulting in a prominent diastolic notch indicating unhealthy vascular status, thus resulting in fewer errors even using general assessment methods. Therefore, the adjustment in SIN identification did not significantly affect the younger (< 50) and older (> 80) age groups, resulting in no change in the proportion of people in these two age groups.

Arterial stiffening is a risk factor for developing CVD and all-cause mortality and is not related to the existence of traditional CVD risk factors [30]. Compared to men, arterial stiffening is almost 2-fold higher in women [31]. It is also known that with the increase in age, women’s pulse pressure and arterial stiffness also increase, and this was independent of aortic size [32, 33]. In addition, common coronary artery dysfunction and heart failure are also related to the increase in arterial stiffness in women [34, 35]. Altogether, significant gender differences have been observed in arterial stiffness-related CVD. This justifies the need to establish a sex-specific upper-limit equation of large artery SI.

Based on the results of our study, SI values and HRV indices derived from PPG signals could be developed to assist in preliminary assessment for SI-related CVD. These indicators can be utilized in wearable devices as vascular health monitoring devices, allowing self-monitoring and awareness of vascular health, and seeking early medical attention if necessary. If the SI value is higher than the general public or the SIN ratio exceeds 80%, with a TP of < 1500 ms², the user could be warned and recommendations on adjustment of lifestyle and dietary habits could be provided to improve vascular health. The extreme specificity of our composite model (100%) effectively “rules in” CVD cases with near-certainty, a crucial feature for preventing unnecessary clinical anxiety and over-diagnosis in remote monitoring scenarios. At the same time, when a larger dataset of SI has been collected, it can be used to further refine the threshold of SI for different subgroups in addition to age, such as individuals with different comorbidities and medical backgrounds.

Despite the promising findings, this study has several limitations that should be acknowledged. First, the potential for sampling bias exists, as participants were recruited through convenience sampling from a specific hospital and university environment. This may not fully represent the general population across different socioeconomic or geographic backgrounds. Second, confounding factors that could affect the output of the PPG signals including individual variations (e.g., obesity), physiological conditions (e.g., body temperature and respiration), and external perturbations (e.g., applied pressure, motion artifact, device efficacy) were not investigated in the present study [36–38]. Third, as an exploratory pilot study, these results lack validation in external or independent cohorts, and the retrospective nature of the analysis precludes the determination of long-term predictive value. Lastly, due to the nature of the single-hospital study, it remains necessary to carry out large-scale, prospective studies to confirm the results of the present study.

## Conclusion

This study provides further evidence for the application of PPG-derived metrics in CV assessment, demonstrating that the proposed three indicators are highly effective in identifying health states. Our findings preliminarily suggest that a multi-parameter approach—combining SI abnormalities, SIN ratio > 80%, and TP < 1500 ms²—may serve as a promising tool for evaluating CVD risk. These results offer a convenient, cost-effective, and automated framework for early CV assessment. Nonetheless, further large-scale prospective studies are warranted to validate these findings and establish clinical generalizability.

## Supplementary Information

Supplementary Material 1.

## Data Availability

The data that support the findings of this study are available on request from the corresponding author.
