# Supplementary material for: Evaluating the efficacy of photoplethysmography-derived stiffness index, sine-waveform ratio, and heart rate variability total power for cardiovascular health assessment
Source: BMC Cardiovasc Disord. 2026 May 9;26:575. doi: 10.1186/s12872-026-05914-6 (PMC13344004; doi:10.1186/s12872-026-05914-6)
Supplement: Supplementary file 1 — Supplementary Material 1. [file 12872_2026_5914_MOESM1_ESM.docx]

**Supplement Figure 1**: An illustration of using the auxiliary line method to identify the presence of sine-waveform. (a) PPG containing diastolic wave; (b) the PPG of the sine wave; (c) first derivative of the PPG in (a); (d) first derivative of the PPG in (b); (e) calculating distance from all points to auxiliary line in (a); (f) calculating distance from all points to auxiliary line in (b).


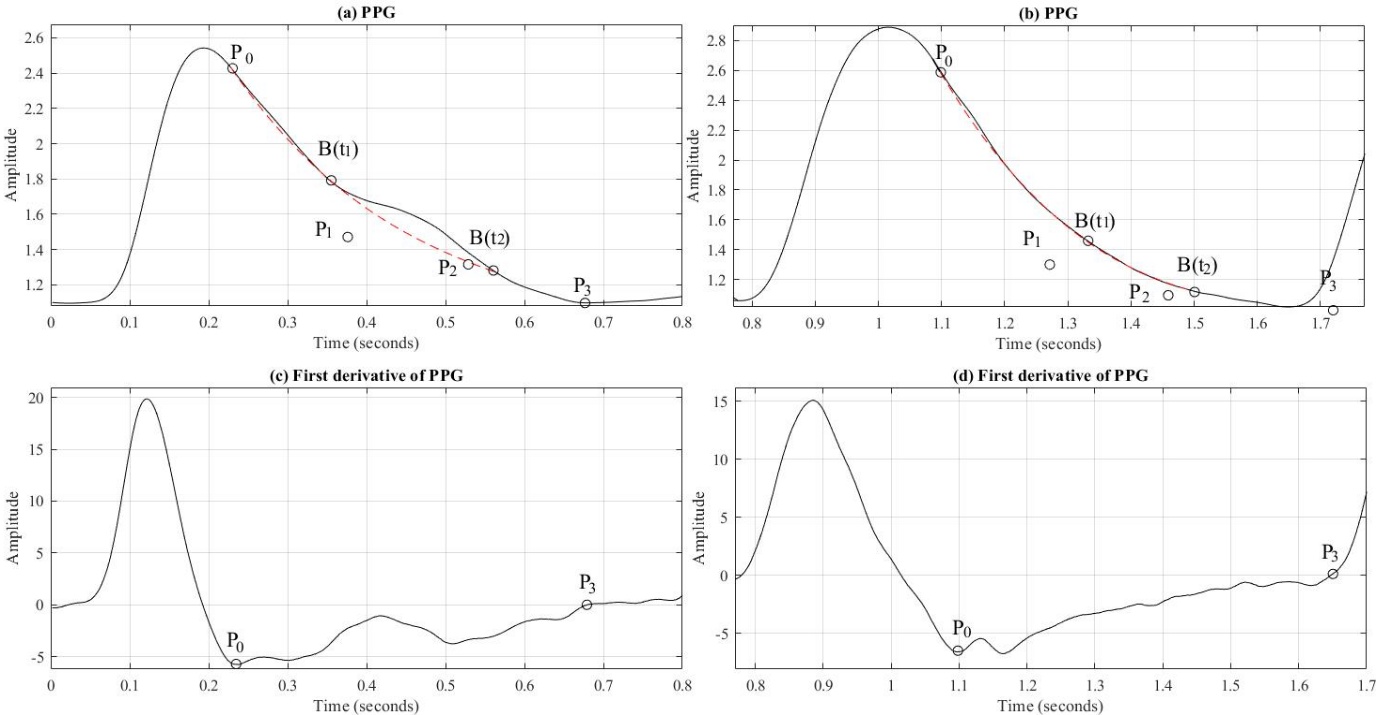


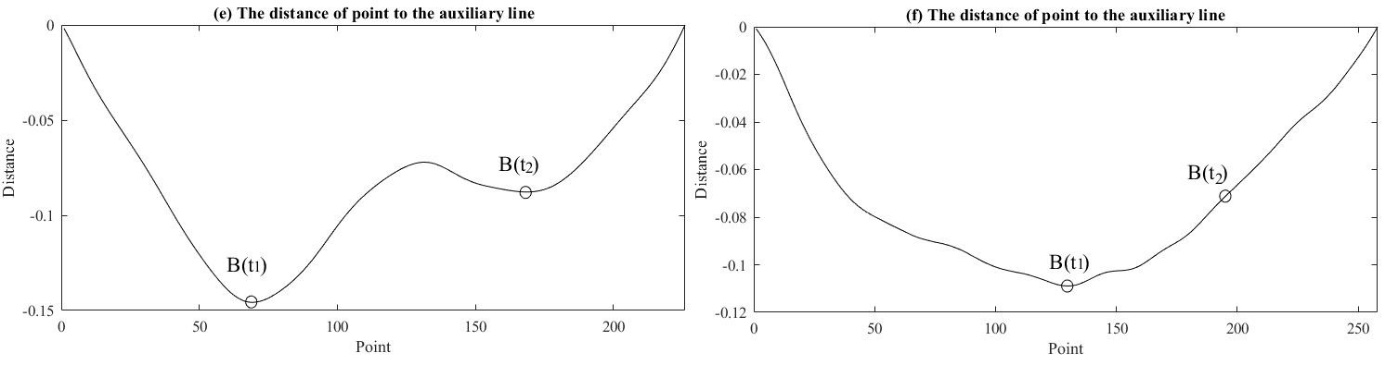


**Supplement Figure 2.** Comparison of total power (TP) value obtained from a 100-second HRV measurement and a standard 5-minute measurement. Data were from MIMIC database (n=100)


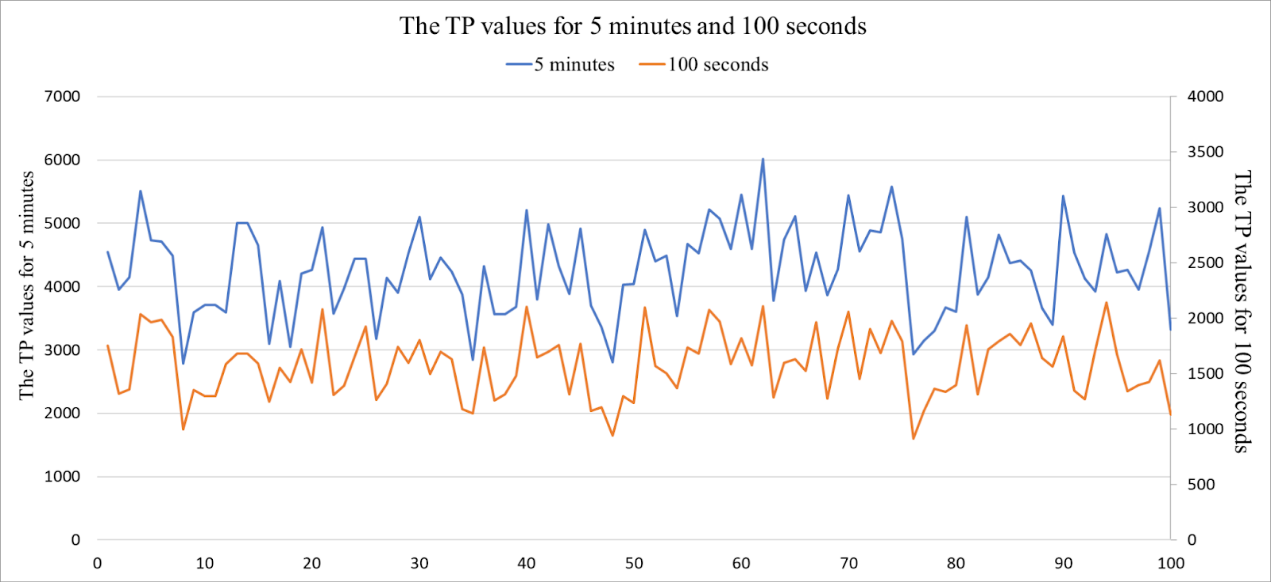


**Supplement Table 1.** The percentage of participants with abnormal TP (<1500 ms^2^) stratified by cardiovascular diseases and age group.

|  | **CVD** | **No CVD** |
| --- | --- | --- |
| Age Group |  |  |
| 1~40 | 0% | 2% |
| 41~50 | 11% | 11% |
| 51~60 | 23% | 13% |
| 61~70 | 38% | 4% |
| 71~80 | 26% | 13% |
| > 80 | 40% | 0% |

CVD, cardiovascular disease; TP, total power is a measure of heart rate variability
